# DECODING DEMENTIA STIGMA THROUGH NETNOGRAPHY: LESSONS FROM THE DEMENTIA DIARIES

**DOI:** 10.1093/geroni/igae098.3182

**Published:** 2024-12-31

**Authors:** Kelly Marnfeldt

**Affiliations:** University of Southern California, Los Angeles, California, United States

## Abstract

Worldwide, 10 million people are diagnosed with dementia each year. The experience of receiving a dementia diagnosis is commonly described in terms of lost identity and self-worth, with feelings of grief, fear of decline, and worries of becoming a burden. Such feelings are exacerbated by pervasive stigmatization and narratives that perpetuate the misconception that a diagnosis equals the end of a meaningful life. Moreover, people with dementia (PWD) are not only absent from stigma-reduction campaigns but they are underrepresented and overlooked across the research-practice-public health continuum. The present study aims to (1) explore perspectives and experiences of dementia-related stigma among PWD and (2) to examine how active participation in awareness and education campaigns by PWD influences their feelings/experiences of stigma. Netnographic methods were employed to analyze archived reflections (~3k) gathered from the UK-based Dementia Diaries—an online platform dedicated to fostering dialogue and combating stigma surrounding dementia. Between 2015 and 2023, 109 individuals contributed 2,946 diary entries to the platform, with 1 to 332 (M=64.04; SD=96.3) entries per person. This analysis identified themes of avoidance, abandonment, and invisibility in PWD’s encounters with stigma, and references to the transformative impact of self-advocacy efforts. PWD attest that involvement in awareness activities fosters humanization and shifts attitudes, promoting inclusion, empathy, and solidarity. Future research should examine the mechanisms underpinning these effects, with an emphasis on personal narratives, advocacy, and stigma-countering strategies. Policy interventions could have broader societal impact by prioritizing meaningful social interactions with PWD and harnessing positive representations in stigma-reduction campaigns.

